# The Various Oximetric Techniques Used for the Evaluation of Blood Oxygenation

**DOI:** 10.3390/s20174844

**Published:** 2020-08-27

**Authors:** Meir Nitzan, Itamar Nitzan, Yoel Arieli

**Affiliations:** 1 Department of Physics/Electro-Optics Engineering, Jerusalem College of Technology, 21 Havaad Haleumi St., Jerusalem 91160, Israel; arieli@jct.ac.il; 2 Monash Newborn, Monash Children’s Hospital, Melbourne 3168, Australia; itamarnitzan@gmail.com; 3 Department of Neonatology, Shaare Zedek Medical Center, Shmuel Bait St 12, Jerusalem 9103102, Israel

**Keywords:** blood oxygenation, oximetry, oxygenated and deoxygenated hemoglobin, light absorption spectra, modified Beer–Lambert Law, oxygen saturation, pulse oximetry, photoplethysmography, near infrared spectroscopy, accuracy

## Abstract

Adequate oxygen delivery to a tissue depends on sufficient oxygen content in arterial blood and blood flow to the tissue. Oximetry is a technique for the assessment of blood oxygenation by measurements of light transmission through the blood, which is based on the different absorption spectra of oxygenated and deoxygenated hemoglobin. Oxygen saturation in arterial blood provides information on the adequacy of respiration and is routinely measured in clinical settings, utilizing pulse oximetry. Oxygen saturation, in venous blood (SvO_2_) and in the entire blood in a tissue (StO_2_), is related to the blood supply to the tissue, and several oximetric techniques have been developed for their assessment. SvO_2_ can be measured non-invasively in the fingers, making use of modified pulse oximetry, and in the retina, using the modified Beer–Lambert Law. StO_2_ is measured in peripheral muscle and cerebral tissue by means of various modes of near infrared spectroscopy (NIRS), utilizing the relative transparency of infrared light in muscle and cerebral tissue. The primary problem of oximetry is the discrimination between absorption by hemoglobin and scattering by tissue elements in the attenuation measurement, and the various techniques developed for isolating the absorption effect are presented in the current review, with their limitations.

## 1. Introduction

Adequate oxygen delivery to tissue is essential for cellular metabolism, in which ATP energy-carrying molecules are produced from oxygen and nutrients. Since oxygen is transported from the lungs to the tissue cells via arterial blood, adequate oxygen supply to a tissue requires sufficient blood flow to the tissue and sufficient oxygen content in arterial blood, CaO_2_ (in mL oxygen per L blood). The latter is related to the hemoglobin concentration in blood, [Hb] (g/L), and arterial oxygen saturation, SaO_2_, (defined as the ratio of oxygenated hemoglobin concentration to total hemoglobin in arterial blood) according to the following relationship [1,2]:

CaO_2_ (mL/L) = 1.36[Hb]SaO_2_ + 0.0031PaO_2_ (1)

where PaO_2_ is arterial oxygen tension (mmHg). The factor 1.36 in Equation (1) stems from the maximal oxygen binding capacity of hemoglobin: 1.36 mL of oxygen in 1 g of hemoglobin. The value of 0.0031 PaO_2_ is the dissolved oxygen content in the arterial blood plasma, and is very small relative to the oxygen content carried by the hemoglobin.

Oxygen saturation, together with concentrations of oxygenated and deoxygenated hemoglobin (HbO_2_ and dHb, respectively), are quantitative parameters that allow the assessment of blood oxygenation. SaO_2_ provides information on the adequacy of respiration and ventilation. Oxygen saturation in venous blood, SvO_2_, also has clinical and physiological significance, because an increase in tissue metabolism requirements or a decrease in blood flow to the tissue lead to greater oxygen extraction by the tissue cells and consequently, to a decrease in oxygen saturation in the blood drained from the tissue by its veins. SvO_2_ is therefore directly related to the blood supply to the tissue that is drained by the vein and inversely related to its metabolism demand. The oxygen saturation in a tissue, StO_2_, is the ratio of oxygenated hemoglobin to total hemoglobin in the entire blood in the tissue—arterial and venous. StO_2_ is directly related to tissue blood flow and inversely related to its metabolism requirements. i.e., StO_2_ provides information on the adequacy of the blood supply to the tissue.

**Figure 1:**
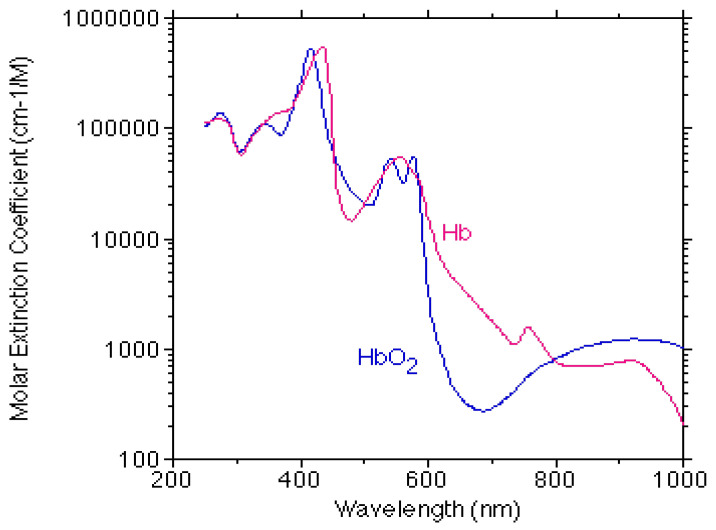
Molar extinction coefficients of oxygenated (blue curve) and deoxygenated hemoglobin (red curve) as a function of the wavelength in the visible and near infrared regions. Prepared by Scott Prahl from a variety of sources [3] and is presented with permission from Dr. Prahl.

Oximetry is a technique for the assessment of blood oxygenation by optical measurements of light transmission through the blood, which is based on the different absorption spectra of HbO_2_ and dHb. Figure 1 presents the molar extinction coefficients of HbO_2_ and dHb (the latter is denoted as Hb in the figure) as a function of the wavelength, in the visible and near infrared wavelength regions [3] (the molar extinction coefficient is defined as the absorption constant per molar and is actually a specific absorption constant). A great difference between the extinction coefficients of HbO_2_ and dHb appears for some wavelengths in the visible region, motivating their use for the oximetric assessment of blood oxygenation. However, light in the visible wavelength region below 650 nm is subjected to substantial absorption, and the transmitted light through tissue might have a low signal-to-noise ratio, even for a tissue with a small optical pathlength, such as the fingertip. Commonly, light in the red and infrared regions is utilized for measurements of light absorption in the fingertip and light in the near infrared region is preferred for measurements of light absorption in thicker tissue. Light in the visible region can also be used for oximetric measurements [4,5,6,7], mainly in the reflection mode.

The assessment of the oxygen saturation and HbO_2_ and dHb concentrations in the arterial and/or venous blood from measurements of light absorption (based on the different absorption spectra of HbO_2_ and dHb) is interfered by the scattering of the light when propagating through the tissue. The optical parameter measured in oximetry is attenuation, the reduction in intensity of light passing through the tissue, and both absorption and scattering contribute to the attenuation. Light is scattered when it is refracted at the border between two media with different refractive indices, such as the surface between intra and extracellular fluids. Scattering contributes to attenuation by the loss of light deflected from its trajectory, and also indirectly, by an increase in the optical pathlength and consequently, an increase in the absorption by the HbO_2_ and dHb molecules. The differentiation between the absorption and scattering components of the attenuation measurement is the primary problem in oximetry and various methods have been developed in the various oximetric techniques for isolation of the absorption component. The scattering constant in tissue and in whole blood monotonically decreases with light wavelength, but the slope is moderate relative to that of the hemoglobin absorption constant. Several studies present curves of reduced scattering coefficient µ_s_’ in tissue [8,9] and in whole blood [10] as a function of light wavelength. µ_s_’ is defined by µ_s_’ = µ_s_(1−g) where µ_s_ is the scattering constant and g is the anisotropy [11,12]. The path that the photon propagates before being scattered is represented by 1/µ_s_; the path the photon propagates before entirely losing its direction is represented by 1/µ_s_’.

The current review presents the main oximetric techniques that have been developed for the evaluation of oxygenation parameters in arterial, venous and tissue blood. The oxygenation parameters are derived from optical attenuation measurements, and the review mainly concentrates on the different approaches for isolating the absorption component from the entire attenuation effect. The degree of differentiation between the absorption and scattering effects is a pivotal determinant for the accurate assessment of oxygen saturation in any oximetric method.

## 2. Oximetry—General Theory

Oximetry is a technique for the evaluation of blood oxygenation level in arteries, veins, or in entire blood in tissue, by optical transmission measurements, utilizing the different absorption spectra of HbO_2_ and dHb. The blood oxygenation parameters are oxygen saturation and HbO_2_ and dHb concentrations in the arterial and/or venous blood. In order to measure the absorption, light with wavelength spectra in the red and/or infrared regions is irradiated into a tissue and its transmission through the tissue is measured. During its passage through the tissue, the light undergoes absorption, mainly by the hemoglobin molecules in the red blood cells, and scattering by tissue elements and the red blood cells. The light absorption that depends on the oxygenation level is the signal and the light scattering that is insensitive to the oxygenation level [10] is a noise, and suitable isolation of the contribution of the absorption to the light attenuation is essential for the accurate evaluation of blood oxygenation level. In order to derive the blood oxygenation from the light absorption measurement, the values of ε_O_ and ε_D_, the extinction coefficients of HbO_2_ and dHb, respectively (Figure 1), are required, and they were obtained by measuring the absorption constant in scattering-free hemoglobin molecule solution, after fragmentation of the red blood cells (hemolysis) [13]. The transmitted light intensity, I_t_, through a sample of homogeneous solution of hemoglobin molecules, is given by the Beer–Lambert law,

I_t_ = I_0_exp(−εCd) (2)

where I_0_ is the intensity of the incident light, d is the width of the hemoglobin solution (the source-detector distance), ε is the hemoglobin solution extinction coefficient and C is the hemoglobin concentration.

The transmitted light intensity, I_t_, through a *scattering tissue* sample, is given by the modified Beer–Lambert law [12,14]:

I_t_ = I_0_exp(−G − εC*l*); ln(I_0_/I_t_) = G + εC*l* (3)

where I_0_ is the intensity of the incident light and *l* is the mean optical path-length. *l* is greater than d because of scattering by the red blood cells and the tissue elements. The ratio *l*/d is named the differential pathlength factor (DPF) and it depends on the light scattering and absorption [12,14]. G represents the light-loss due to tissue scattering and depends on the light-sources and detector geometry. In the transmission mode of measurement, G represents the light-loss due to light deflection from a straight-line trajectory, while in the reflection mode (where the light-source and the detector are positioned on the same side of the tissue sample), G is more complex, as scattering also enables the diversion of light towards the detector.

The weighted mean extinction coefficient ε of the blood in the vessels of the tissue sample depends on the oxygen saturation SO_2_ in the tissue blood:

ε = ε_O_SO_2_ + ε_D_(1 − SO_2_). (4)

Combining Equations (3) and (4), a linear relationship between the physiological parameter SO_2_ and the measured parameter ln(I_0_/I_t_) can be obtained:

ln(I_0_/I_t_) = G + εC*l* = G + ε_D_C*l* + C*l*(ε_O_ − ε_D_) SO_2_. (5)

C, (in units of molar), is either the hemoglobin concentration in tissue, when the measurement is done on blood in tissue, or the hemoglobin concentration in a blood sample in a blood vessel. If light is transmitted through a tissue, other substances, such as melanin, myoglobin and cytochrome, might also affect the absorption.

In order to derive SO_2_ from measurement of ln(I_0_/I_t_) through Equation (5), the three unknowns, G, C and *l*, should be considered. The two effects of scattering, the light deflection and the lengthening of the optical path-length, considerably affect the light attenuation and prevent quantitative derivation of oxygen saturation from light transmission measurements. In addition, G and DPF change considerably among different persons, tissues and situations, and quantitative measurement of them in each examination is impractical. In oximetry, transmission measurement, in two or three wavelengths, has been utilized to reduce the harmful effect of the scattering in oximetry. The benefit of two-wavelength oximetry arises from the considerable spectral dependence of the difference in extinction coefficient between HbO_2_ and Hb, while the spectral changes in scattering are moderate [8,9,10].

## 3. Non-Pulsatile Oximetric Techniques

### 3.1. In-Vitro Measurements

Early in vitro oximetric measurements of oxygen saturation in whole blood (blood that contained intact red blood cells) were based on the assumption that light absorption and scattering can be treated independently—the theory of Twersky [15,16,17]. Then, the modified Beer–Lambert law can be written as OD = G + εcd, where OD is the optical density (OD = log_10_(I_0_/I_t_) = ln(I_0_/I_t_)/2.3). In this equation, the source-detector distance, d, was used as the pathlength (instead of *l* in Equation (3)), neglecting the effect of scattering by red blood cells on pathlength elongation.

Pittman [16] isolated the contribution of scattering to attenuation by measuring the optical density at two wavelengths (546 and 520 nm), for which the extinction coefficients of dHb and HbO_2_ were equal (isosbestic wavelengths) and their scattering effect was similar, because they were close to each other. SO_2_ could be obtained from an additional measurement of the optical density at a third wavelength (555 nm) in which the extinction coefficients of Hb and HbO_2_ showed a large difference. SO_2_ was determined from the corrected optical densities at the wavelengths 555 nm and one of the isosbestic wavelengths: (OD(555)-G)/(OD(520)-G). The three-wavelength technique with two isosbestic wavelengths was also used later in retinal oximetry [18,19,20], dealt with in Section 4.3.

**Figure 2:**
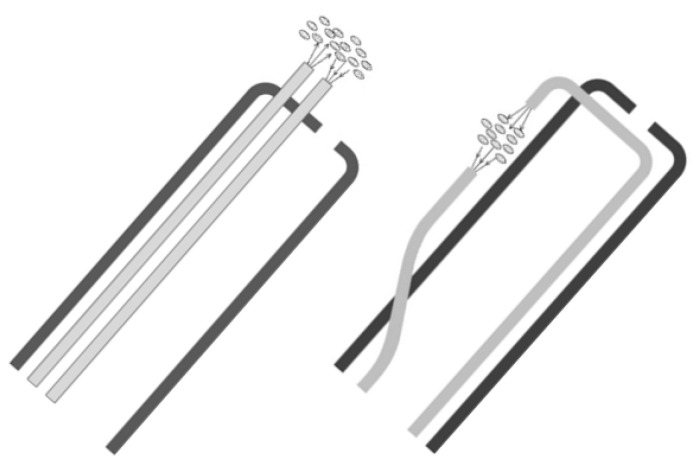
Reflection (**left**) and transmission (**right**) fiber-optic intravenous blood oximeters. The emission and measuring optic fibers are drawn in grey, the catheter wall is drawn in black.

Iwasaki et al. [17] measured the oxygen saturation of whole blood collected from rabbits by means of a fiber-optic catheter inserted in the blood (see Figure 2). They used two wavelengths, an isosbestic one of 805 nm, and another of 670 nm, for which the extinction coefficients of dHb and HbO_2_ showed a great difference. Based on the theory of Twersky, they employed an expression:

SO_2_ = (k_1_/C_Hb_)(OD_670_-OD_805_) + k_2_ (6)

where C_Hb_ is the total hemoglobin concentration and k_1_ and k_2_ are constants that depend on the catheter geometry and hemoglobin extinction coefficients. OD_670_ and OD_805_ are the measured optical densities at 670 nm and 805 nm, respectively.

Equation (6) was validated in-vitro on a blood sample, making use of SO_2_ measured by a commercial oximeter (Radiometer), which employed two wavelengths in the visible range, 505 nm and 600 nm. The correlation between the two oximetric techniques was 0.99 (*p* < 0.001) for a saturation range of 13–100%, with standard deviation of 1.1%.

### 3.2. Tissue Oximetry

An oximeter that utilized red and green light transmission through the outer ear was developed during World War II [21]. Red light is more sensitive to the hemoglobin oxygenation level than broadband green light (Figure 1). The study included a series of examinations, each comprised an initial calibration by comparing in vivo measurement during pure oxygen breathing, and measurement on extracted arterial blood with 100% saturation. Then, in vivo measurements were performed, in which the oxygen level in the inspired air was lowered. The calibration enabled the elimination of the scattering effects and the effect of light absorption by venous blood. The accuracy of the device, as determined by gas analysis of arterial blood samples, was 3–8%.

An ear oximeter that did not require calibration in each examination, but only pre-calibration, was developed by Hewlett-Packard in the 1970’s, (at about the same time as that of pulse oximetry invention), using eight wavelengths, from 650 nm to 1050 nm. The ear was chosen because its high blood flow is in excess of its metabolic needs, as it serves as a heat radiator. The coefficients required for the determination of arterial oxygen saturation from the optical measurements were derived by pre-calibration, using simultaneous measurements of light transmission and oxygen saturation in arterial blood extracted from volunteers that were performed in different oxygen saturation values, obtained by breathing different gas mixtures [22,23,24].

The calibration process could take account of the contribution of non-hemoglobin tissue absorbers and the scattering by the tissue and red blood cells. In order to compensate for changes in venous blood concentration, ear temperature was increased to 41 °C (or vasodilator cream was applied to the ear), “arterializing” the venous and capillary blood. The Hewlett-Packard eight-wavelength oximeter was compared to another oximeter (Radiometer), and showed a standard error of estimate of 2–2.5% [22,23,24].

The Hewlett-Packard oximeter has not been produced since 1983 [25], because the pulse oximeter that was invented in 1972 by Takuo Aoyagi [26] has been found to be more suitable for clinical practice. In a subsequent section, it will be described how pulse oximetry, after pre-calibration, solved the problems of interference of scattering and absorption in venous blood in a more practical way.

### 3.3. Intravenous Blood Oximetry

As described in the Introduction section, the oxygen saturation in a vein’s blood, SvO_2_, is related to the blood supply to the tissue that is drained by the vein. Of particular significance is the oxygen saturation in the pulmonary artery that carries the mixed venous blood—the entire venous blood returning from the body organs supplied by the systemic circulation—and in the jugular veins, which drain the blood from the brain. SvO_2_ in the pulmonary artery and in the jugular vein are directly related to the cardiac output and brain blood supply, respectively, and inversely related to the oxygen demand in the respective tissue.

Mixed venous oxygen saturation in the pulmonary artery can be obtained by introducing a Swan–Ganz catheter into the pulmonary artery and measuring the oxygen saturation in an extracted sample of mixed venous blood via blood gases analysis. Pulmonary artery catheters are introduced through a central vein, the right atrium and ventricle, and because their use has been limited, measurement of oxygen saturation in central veins—superior and inferior vena cava—has been suggested as a surrogate for mixed venous oxygen saturation measurement [27,28,29].

Monitoring oxygen saturation by intermittent measurement in extracted blood samples has several drawbacks, and intravenous blood oximetry has been developed for continuous monitoring of oxygen saturation in veins’ blood. The oximetric probe consisted of two optical fibers with a small gap between their tips (Figure 2), which was introduced into the vein. One of the fibers transmits two-wavelengths of light into the blood in the gap between the optical fibers and the second fiber guides the transmitted light to the detector [17,29,30].

The SvO_2_ value was calculated from the values of the transmitted light in two or three wavelengths by using the modified Beer–Lambert law [17,30]. Like the Millikan’s oximeter, the intravenous oximeter needs calibration in a blood sample *before each continuous examination*, according to the manufacturer’s instructions, using blood gases analysis. Limits of agreement of 7–16% were found between the optical measurement and blood gases analysis on blood samples extracted from the same vein [28,29].

Besides its use in the central veins, the intravenous blood oximeter has also been implemented for the measurement of oxygen saturation in the jugular vein, which drains blood from the brain. However, while low SvO_2_ in the jugular vein’s blood faithfully indicates inadequate oxygen supply to the brain, normal values of jugular oxygen saturation cannot ascertain adequate oxygen delivery to each brain site, because insufficient oxygen supply to a small region of the brain might only slightly affect SvO_2_ in the jugular vein, which drains blood from the entire brain hemisphere [31,32]. Several studies showed an association between abnormalities in jugular SvO_2_ and poor outcomes in patients after traumatic brain injury [33,34,35].

## 4. Pulse Oximetry—Theory and Techniques

### 4.1. Theory of Arterial Pulse Oximetry

The pulse oximeter, which measures the oxygen saturation in the arterial blood, SaO_2_, was invented in 1972 by Takuo Aoyagi, an electrical engineer at the Nihon Kohden company in Tokyo [26]. Pulse oximetry evaluates the SaO_2_ level by optical transmission measurements in two wavelengths, utilizing the different absorption spectra of HbO_2_ and dHb, like the other oximetric techniques. In order to measure oxygen saturation only in the arterial blood, the light absorption is measured selectively in the systolic increments of arterial blood volume after ventricular contraction, by recording heart-induced oscillations in light absorption—photoplethysmography (PPG).

The theory of pulse oximetry has been described in several publications [36,37,38,39,40]. In the current review, the theory and its assumptions will be presented in detail, in order to demonstrate its limitations and its relationship to the other oximetric techniques.

**Figure 3:**
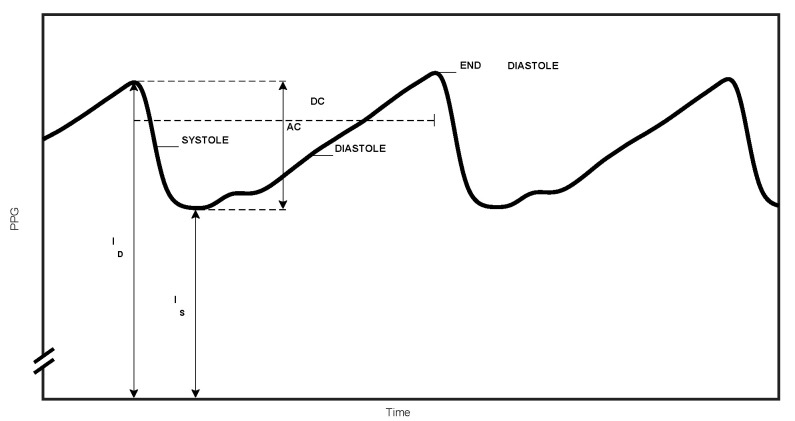
The measured parameters of the PPG pulses. The transmitted light through the tissue decreases during systole and increases during diastole. AC is the difference between the maximal (I_D_) and minimal (I_S_) light transmission through the tissue; DC is the mean light transmission during the pulse. This Figure was published in Yossef Hay et al. Sensors 2018 [41].

A finger PPG signal is shown in Figure 3, presenting the decrease in transmitted light intensity through the finger during systole and the light intensity increase during diastole, due to the systolic increase and diastolic decrease in the arterial blood volume. The maximal and minimal values of the PPG pulse (I_D_ and I_S_ in Figure 3, respectively) are proportional to the light transmission through the tissue at end-diastole and at end-systole, when tissue blood volume is minimal and maximal, respectively. The analysis of the PPG pulses enables the isolation of the arterial blood contribution to the entire light absorption.

Substitution of the I_D_ and I_S_ values for I_t_ in Equation (3), and division of the resultant two equations, eliminate the value of I_0_, and provide an equation for I_D_/I_S_:

I_S_ = I_D_exp(G_D_ + εC_D_*l*_D_)exp(−G_S_ − εC_S_*l*_S_); ln(I_D_/I_S_) = G_D_-G_S_ + εC_D_*l*_D_ − εC_S_*l*_S_ (7)

The D or S subscripts indicate the parameter values at diastole or systole, respectively. G_D_, G_S_, *l*_D_ and *l*_S_ are unknown, but it can be assumed that the changes in G and *l* due to the systolic arterial blood increment can be neglected relative to the absorption effect (G_D_ ≈ G_S_, *l*_D_ ≈ *l*_S_ ≈ *l*). Accordingly, Equation (7) can be written as:

ln(I_D_/I_S_) = εΔC*l* (8)

The PPG amplitude, I_D_–I_S_, is generally small relative to I_S_, and Equation (8) can be written as:

(I_D_-I_S_)/I_S_ = εΔC*l* (9)

where ΔC is the maximal hemoglobin concentration increase in the tissue during systole, due to the arterial blood volume increase. ε is the extinction coefficient for the arterial blood, which includes both oxi- and deoxi-hemoglobin, and depends on the oxygen saturation in the arterial blood, SaO_2_.

When light transmission is measured in two wavelengths, λ_1_ and λ_2_, Equation (9) becomes:

[(I_D_ − I_S_)/I_S_]_1_ = ε_1_ΔC_1_*l*_1_; [(I_D_ − I_S_)/I_S_]_2_ = ε_2_ΔC_2_*l*_2_ (10)

In pulse oximeters, the light in two wavelengths is emitted from two adjacent light-emitting diodes (LEDs) and the transmitted light is measured by a single detector. Since the light of the two wavelengths propagates through the same tissue element, the blood concentration change, ΔC, in the measurement site for the two wavelengths can be considered equal (ΔC_1_ ≈ ΔC_2_).

By dividing the equations in Equation (10) one gets:

(11) $$R\equiv \frac{{\left[\left(I_{D}-I_{S}\right)/I_{S}\right)}_{1}}{{\left[\left(I_{D}-I_{S}\right)/I_{S}\right)}_{2}}=\frac{\varepsilon_{1}l_{1}}{\varepsilon_{2}l_{2}}$$

If we further assume that *l*_1_ is not much different than *l*_2_, then

R ≈ ε_1_/ε_2_ (12)

Substitution of Equation (4) in Equation (12) provides a relationship between the measured parameter R and the physiological parameter SaO_2_:

(13) $$R=\frac{\varepsilon_{D}{}_{1}+{\mathrm{SaO}}_{2}\left(\varepsilon_{O1}-\varepsilon_{D1}\right)}{\varepsilon_{D1}+{\mathrm{SaO}}_{2}\left(\varepsilon_{O2}-\varepsilon_{D2}\right)}$$

and

(14) $${\mathrm{SaO}}_{2}\;=\;\frac{\varepsilon_{D1}-{R\varepsilon }_{D2}}{R\left(\varepsilon_{O2}-\varepsilon_{D2}\right)+\left(\varepsilon_{D1}-\varepsilon_{O1}\right)}$$

Equation (14) could provide the required relationship between SaO_2_ and R, since the values of ε_O_ and ε_D_, the extinction coefficients for HbO_2_ and dHb, are known for wavelengths in the red and infrared regions [42,43]. However, the assumption that *l*_1_ is not much different than *l*_2_ (Equation (12)) can introduce significant error in the calculation of SaO_2_, when the two wavelengths differ significantly, since the optical path-length increases by light scattering and the scattering constant decreases monotonically with the wavelength [8,9,10]. In the commercial pulse oximeters that make use of two wavelengths in the red and infrared regions, a non-negligible difference in optical path-lengths between the two wavelengths is expected.

If the difference between *l*_2_ and *l*_1_ is not neglected, Equation (12) should be modified to

R(*l*_2_*/l*_1_) = ε_1_/ε_2_ (15)

Furthermore, by replacing R in Equation (14) with R(*l*_2_/*l*_1_), the relationship between SaO_2_ and R becomes [39,41,44]:

(16) $${\mathrm{SaO}}_{2}\;=\;\frac{\varepsilon_{D1}-R\left(l_{2}/l_{1}\right)\varepsilon_{D2}}{R\left(l_{2}/l_{1}\right)\left(\varepsilon_{O2}-\varepsilon_{D2}\right)+\left(\varepsilon_{D1}-\varepsilon_{O1}\right)}$$

Generally, the factor *l*_2_*/l*_1_ is not known with reasonable accuracy and SaO_2_ cannot be derived from the measured value of R by Equation (16). In practice, SaO_2_ is obtained from the measured parameter R by calibration, for each kind of pulse oximeter sensor [37,38,45,46]: R is measured for a number of subjects simultaneously with in vitro SaO_2_ measurement in extracted arterial blood by means of a co-oximeter (the gold-standard for SO_2_ measurements). For each person, R and the corresponding SaO_2_ are measured for several values of oxygen partial pressure in the inspired air, and a look-up table is prepared from the R-SaO_2_ pairs. In clinical examinations, SaO_2_ is determined from the R value through an empirical function that was derived from the look-up table. The calibration enables the determination of SaO_2_ from the R measurement only, while the use of Equation (16) must utilize the value of *l*_2_/*l*_1_, which is not known with adequate precision.

Note that in general, in publications about pulse oximetry, the ratio of ratios, R, is defined by means of the pulsatile and non-pulsatile (average) components of the detected PPG pulse: R ≡ (AC/DC)_1_/(AC/DC)_2_, which seems to be different from the definition of R in Equation (11) (See Figure 3 for the definitions of AC and DC). However, AC is equal to I_D_ − I_S_ and DC is the average value of the PPG pulse during the cardiac cycle. DC is only slightly greater than I_S_, assuming that I_D_ − I_S_ is small relative to I_S_.

The validity of the clinical measurements by the calibrated pulse oximeter is based on the assumption that the extinction coefficients and *l*_2_/*l*_1_ do not change significantly between different persons and different physiological and clinical situations. While the HbO_2_ and dHb extinction coefficients for a given wavelength are invariable, the scattering by tissue and red blood cells strongly depends on the substance and structure of the tissue and on the volume and anatomical arrangement of blood vessels in the tissue, which significantly varies among patients. It is expected that *l*_2_/*l*_1_ in a specific clinical examination might deviate from the *mean l*_2_/*l*_1_ value for the persons examined in the calibration process, and that deviation is probably the main origin of the inaccuracy in the assessment of SaO_2_ by pulse oximetry (See below). For wavelengths in the red and infrared regions, the differences between their scattering constants and pathlengths are great [8,9,10] and accordingly, the absolute variation in the pathlengths ratio, *l*_2_/*l*_1_, among patients is expected to be considerable.

The actual mean error in SaO_2_ measurement by the available commercial pulse oximeters is 3–4% for adults and 4–6% for newborns [47,48,49,50,51,52,53,54]. In the low SaO_2_ levels, below 80 or 90%, the error is even greater [50,51,53]. Because of its low accuracy, arterial oxygen saturation measurement by pulse oximetry is denoted by SpO_2_, while the term SaO_2_ is generally reserved for arterial oxygen saturation measured by blood gases analysis in extracted blood. The accuracy in SaO_2_ measurement is of particular importance in preterm newborns treated with oxygen supplementation, because low oxygenation increases mortality while excessive oxygenation increases the risk of retinopathy of prematurity [53,55,56]. Accurate SaO_2_ measurement is also important in adult patients in intensive care units, treated with high concentrations of inspired oxygen, where hyperoxia is associated with greater mortality and morbidity [57,58].

In the last few years, pulse oximetry that makes use of two close wavelengths in the infrared has been developed [39,41,59]. The choice of light with two close wavelengths reduces the differences between their scattering constants and path-lengths, so that the error due to the inter-subject variation in *l*_2_/*l*_1_ is expected to be small relative to that experienced for red and infrared pulse oximetry. In those studies, SpO_2_ was derived from Equation (14), using the values of the extinction-coefficients of HbO_2_ and dHb, as presented in the articles of Kim and Liu [42,43], and neglecting the difference between *l*_2_ and *l*_1_. In the earlier study [39], simultaneous examinations of SaO_2_ were performed with two close wavelengths in the infrared region (767 and 811 nm) and two wavelengths in the red and infrared regions (635 and 937 nm), making use of Equation (14). The discrepancy of the results from a commercial pulse oximeter (that was calibrated by extracted blood) was 3% for the two-infrared wavelengths pair and 9% for the red–infrared pair, probably due to the greater deviation of the ratio *l*_2_/*l*_1_ from the value of 1 in the red-infrared pair. The bottom line is that the two-infrared pulse oximeter also needs calibration, but since the difference between *l*_2_ and *l*_1_ is small, the variability in *l*_2_*/l*_1_ for the two-infrared pair of wavelengths is expected to be smaller than that for two wavelengths in the red and infrared regions.

In a recent study [41] on newborns and children with an arterial line, simultaneous measurements of SpO_2_, using two infrared wavelengths and SaO_2_ by blood gases analysis on extracted blood, were performed. The mean difference between SaO_2_ and SpO_2_ was 3%, which was corrected by multiplying the SpO_2_ results by a calibration factor of 1.03. Compared to arterial line measurements, the standard-deviation of the two-infrared-wavelength SpO_2_ measurements was 3.3%, similar to that of a commercial pulse oximeter (3.8%).

### 4.2. Pulse Oximetry of Venous Blood

Oxygen saturation in venous blood has been measured by modified pulse oximetry, in which a pulsatile increase in venous blood volume was achieved either by applying pressure proximal to the measurement site—e.g., a finger [39,60]—or by mechanical ventilation [61,62]. The latter technique is based on the phenomenon that mechanical ventilation induces systemic blood volume changes and on the assumption that those changes are of venous origin. SvO_2_ was measured by Walton et al. on cardiac surgery patients making use of an esophageal PPG probe, and by Wolf et al. on cerebral tissue of infants. The two studies yielded saturation values around 80% [62] or 70% [61], which are within the physiologic range of SvO_2_ in the relevant tissues.

In the two other studies [39,60], external pressure of 40–50 mmHg was applied proximal to the measurement site, leading to an increase in venous blood volume, and two-wavelength light absorption was used for the measurement of SvO_2_. Nitzan et al. [39] used a single step increase in the cuff pressure and two close wavelengths in the infrared region in order to reduce the difference in their pathlengths (see end of Section 4.1). In the study of Khan et al. [60], artificial venous blood volume oscillations at a frequency of 0.2 Hz were induced by a pressure cuff, placed on the finger, proximal to a commercial pulse oximeter sensor. The interquartile range of the SvO_2_ difference between the venous pulse oximeter and a reference blood gas analyzer was −2.05–1.27%.

### 4.3. Difference Oximetry in Single Arteries and Veins

In pulse oximetry, the measurement of the attenuation difference between end-systole and end-diastole, given by ln(I_D_/I_S_), enables the determination of SaO_2_, by assuming that the difference in light-loss due to scattering between end-diastole and end-systole can be neglected relative to the absorption effect. In a similar way, difference oximetry was suggested for oxygen saturation measurements in a single blood vein, by introducing light in two wavelengths into the vein and measuring light reflection from two nearby sites in the vessel [63]. The method was found feasible by Monte Carlo simulation of light backscattering from a vein model.

**Figure 4:**
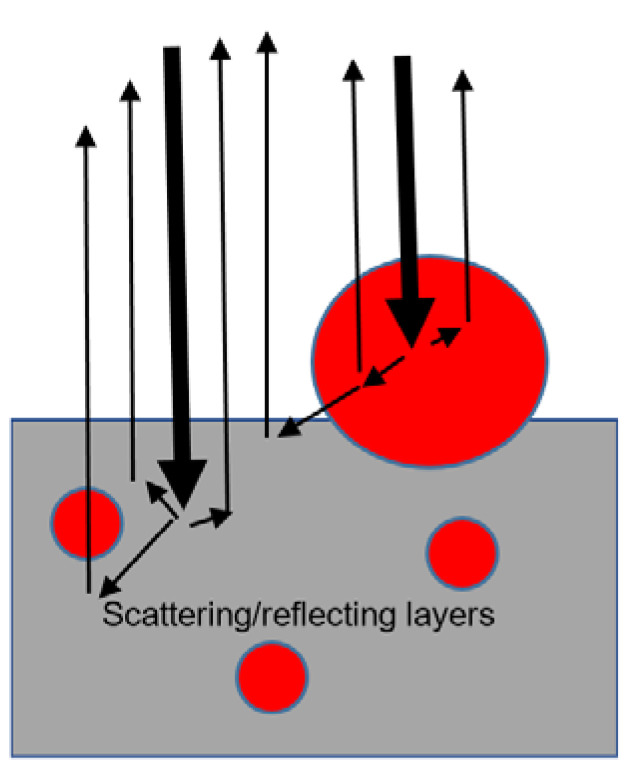
Schematic cross-section of the retina (grey), which includes cross-sections of a retinal vessel (arteriole or venule) and retinal capillaries (red), and possible trajectories of light that illuminates the retina. The incident light (broad black rays) illuminates the vessel and its neighborhood. The light that strikes the vessel and the neighboring tissue is scattered and backscattered by the blood and the tissue (thin rays). Most of the scattered/reflected light from the neighboring tissue is not affected by absorption in the blood, but some photons are affected by blood in the retinal vessel or the retinal capillaries.

In practice, difference oximetry has been implemented in oxygen saturation measurements in single retinal arteries and veins in the retina, by illuminating the eye fundus and acquiring its image through the eye’s pupil, mostly with a fundus camera, exploiting the eye’s transparency. Figure 4 presents light rays’ trajectories from the eye’s lens to the retina and scattered/reflected light from a retinal blood vessel and its neighborhood (See Hammer et al. [64]). Most of the light that reaches the retina is lost by scattering or absorption in blood vessels, but a small part of the scattered/reflected light is recorded by the camera. Retinal image analysis enables discrimination between scattered/reflected light from a blood vessel (I_v_) or from its neighborhood (I_0n_). (Note that in retinal oximetry studies, scattered light from the vessel’s neighborhood is denoted by I_0_. We changed the symbol; as in the Beer–Lambert law, I_0_ denotes the incident light, see Equations (2), (3) and (5)) Retinal oximetry is based on the assumptions that I_0n_ is unaffected by light absorption in the blood and the light-loss G due to scattering is similar for the two measurement sites, in analogy to pulse oximetry that assumes similarity in light-loss G for end-systole and end-diastole (see discussion after Equation (7)). Hence, the difference between I_v_ and I_0n_ is assumed to stem from the light absorption in the vessel’s blood, and the ratio between the optical densities for the two wavelengths enables the determination of oxygen saturation in the retinal vessel. It seems, however, that the assumption that I_0n_ is unaffected by light absorption in the blood is not fully justified, as can be inferred from Figure 4.

The optical density of the vessel (OD_Ves_) is defined as,

OD_Ves_ = log(I_0n_/I_v_) (17)

reflecting the effect of light absorption in the vessel’s blood. In practice, OD_Ves_ is obtained along the image of the vessel and next to it. The retinal image is split into two images created with two wavelengths in the visible region: λ_1_, in which the extinction coefficient substantially differs between HbO_2_ and dHb, and λ_2_ that is insensitive to oxygen saturation—an isosbestic wavelength. The image separation is performed either by an image splitter and appropriate filters [65,66], a double bandpass filter and the red and green camera channels [67,68], or a liquid crystal tunable filter [69]. The ratio between OD_Ves_ in the two wavelengths, OD_Ves1_/OD_Ves2_, is the measured parameter, from which the oxygen saturation is derived, assuming a linear relationship between the OD_Ves1_/OD_Ves2_ and SO_2_ (either SaO_2_ or SvO_2_),

SO_2_ = a + k(OD_Ves1_/OD_Ves2_). (18)

a and k are constants that were determined by calibration, in which SO_2_ was measured in systemic arteries by a pulse oximeter, simultaneously with the retinal arteries examination, during varied hypoxia levels, obtained by breathing different oxygen concentrations [65]. In other studies [66,67], these constants were determined by comparing OD_Ves1_/OD_Ves2_ values in retinal arteries or veins to the mean of retinal SaO_2_ or SvO_2_ data, obtained in former studies [18,20]. Here, the ratio OD_Ves1_/OD_Ves2_ plays the same role as the ratio-of-ratios R in pulse oximetry (See Equations (8), (9) and (11)).

One of the two wavelengths in the visible region that are used in retinal oximetry is a wavelength in which the dHb and HbO_2_ extinction coefficients have a large difference, such as 600, 610 or 555 nm, and the other is 548 or 570 nm [67,68,70], an isosbestic wavelength in the 500–600 nm range [19]. In a number of studies [18,19,20], retinal SO_2_ was obtained by measuring scattered/reflected light with three wavelengths in the visible region, making use of the method of Pittman and Duling [16], which was mentioned in Section 3.1.

#### Accuracy and Applications

The accuracy of retinal oximetry can be evaluated by comparing SaO_2_ values, measured in retinal arteries, to systemic SaO_2_ values, measured in the peripheral arteries either noninvasively by pulse oximetry or by co-oximeter in extracted arterial blood. The comparison is based on the assumption that SaO_2_ is constant throughout the entire systemic arterial system, as oxygen diffuses from blood vessels into tissue only in the capillaries.

Comparison of retinal SaO_2_ readings to systemic SaO_2_ values was primarily done with two commercial retinal oximeters: The Dynamic Vessel Analyzer (DVA, Imedos, Jena, Germany) and the Oxymap (Oxymap, Reykjavik, Iceland). The mean of the retinal SaO_2_ readings by Oxymap, measured in healthy persons, was lower by 5% than finger SaO_2_, measured by pulse oximetry, with 95% CI of −2 to 12% [71]. Measurements of mean retinal SaO_2_ in patients with systemic hypoxemia were lower by 2% with 95% CI of −3 to 11% compared to invasive femoral SaO_2_ values [72]. No significant correlation was found between individual retinal and finger SpO_2_ [72,73,74]. In a study performed with the DVA retinal oximeter on chronic obstructive pulmonary disease (COPD) patients [75], a low correlation (r = 0.6, *p* < 0.05) was found between retinal and finger SpO_2_ readings, and a Bland–Altman plot, comparing retinal and finger SpO_2_ data, yielded 1.96SD of ±10%. Told et al. [76] found that the individual retinal SaO_2_ readings, measured by the DVA and Oxymap retinal oximeters, were significantly different.

**Figure 5:**
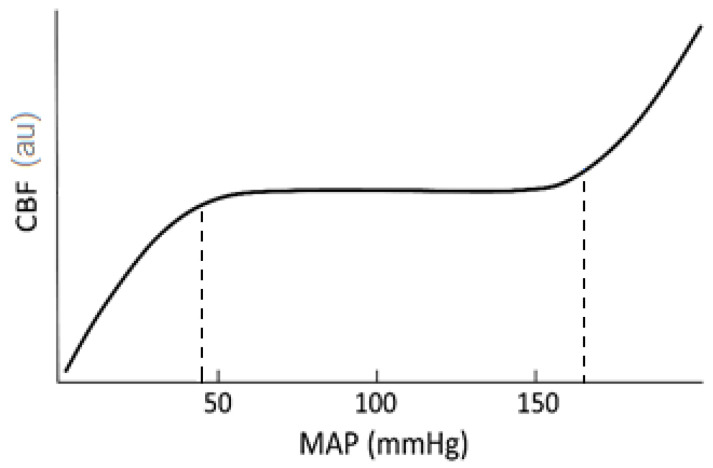
Schematic presentation of the relationship between cerebral blood flow CBF and mean arterial pressure MAP, affected by autoregulation. The autoregulation is only effective between two MAP limits, presented by dashed lines. For MAP lower than the left limit, the brain tissue is ischemic, and for MAP greater than the right limit, the brain tissue is hyperemic, potentially leading to edema with its adverse consequences.

The lower mean retinal SaO_2_, relative to finger SpO_2_, was explained by an error in the process of the retinal oximeters’ calibration or by the countercurrent exchange of oxygen by diffusion from the arteries within the optic nerve [70,76,77]. The low value of retinal SaO_2_ can also be attributed to the measurement of I_0n_, the scattered light from the blood vessel neighborhood, which might be affected by light absorption in retinal blood vessels (See Figure 5).

The simultaneous measurement of oxygen saturation in a pair of retinal artery and vein can assess the arteriovenous difference of oxygen saturation and oxygen content, CaO_2_-CvO_2_ (See Equation (1)). The latter provides information on the retinal oxygen consumption in the tissue supplied by the artery and drained by the vein, an important parameter, which is related to the metabolism rate of the retinal cells [78,79]. The arteriovenous difference of oxygen saturation or oxygen content decreased during breathing of 100% oxygen relative to breathing air in measurements on healthy subjects [78,80], but was unchanged in COPD patients [75].

Non-invasive retinal oximetry has been used in physiological studies on healthy persons [68,70,74,78,79,80] and on patients with ophthalmic and a number of systemic diseases [70,75,81,82,83,84,85,86]. The increase in arteriovenous difference of retinal oxygen saturation is generally due to ischemia, while the decrease in that difference might originate from atrophy, which is associated with lower metabolism of retinal cells. Ischemic diseases include diabetes, central retinal vein occlusions, age-related macular degeneration and retinopathy of prematurity. Atrophy-related retinal diseases include glaucoma and retinitis pigmentosa [70].

The accessibility to light of the human retina has been utilized as a window to the brain, as the retina and its optic nerve are considered an extension of the central nervous system, from the anatomic, functional and pathologic aspects [87,88,89]. Like the brain, the retina has a large demand for oxygen and nutrient supplies [88], and both tissues utilize autoregulation to keep sufficient blood supply [90]. Retinal oxygen saturation has been suggested as a mirror to brain oxygenation. In studies on mild cognitive impairment and multiple sclerosis patients [91,92], retinal arteriovenous oxygen saturation difference was significantly lower than that of healthy persons, which reflects reduced oxygen extraction in the retina, indicating reduced metabolism.

## 5. Near Infrared Spectroscopy (NIRS)

### 5.1. NIRS Techniques

Near infrared spectroscopy (NIRS) is an oximetric technique that provides assessment of the HbO_2_ and dHb concentrations ([HbO_2_] and [dHb], respectively), and of oxygen saturation in the entire blood in tissue—arterial, venous and capillary. The tissue oxygen saturation, StO_2_, is also named as the tissue oxygenation index, TOI, or regional oxygen saturation, rSO_2_. Like other oximetric techniques, NIRS is based on the different absorption spectrum of HbO_2_ and dHb and the measurement of light attenuation in two or more wavelengths. As an optical technique, it can non-invasively measure oxygenation in muscles and cerebral tissue that are accessible to light, since infrared light can penetrate several centimeters in tissue [93,94]. In order to non-invasively assess oxygenation or oxygenation change in the cerebral tissue, light is emitted into the brain through the intact skull and the light transmitted through the cerebral tissue is measured by a detector located 2–4 cm from the light-source. The greater the distance between a pair of optodes (the light-source/detector pair), the deeper cerebral tissue examined. For a typical differential pathlength factor (DPF, the ratio between mean actual pathlength and optodes-distance) value of 5, a distance between optodes of 2–4 cm is equivalent to a transmitted light pathlength of 10–20 cm. Because of the long pathlength, the wavelengths utilized in NIRS are in the near infrared region, 700–1000 nm, where light absorption is relatively small, see Figure 1. Wavelengths above 1000 nm are significantly absorbed by water [95,96].

In the near infrared region, the main absorbers are HbO_2_ and dHb. The enzyme cytochrome oxidase (CytOx) is also involved in light absorption in cerebral tissue in this wavelength range, but its concentration is much less than the hemoglobin molecules [97]. CytOx is an enzyme in the mitochondrial electron transport chain that synthesizes the adenosine triphosphate (ATP)—the molecule that provides the energy required for cellular metabolism. Since CytOx oxygenated and deoxygenated states differ in their infrared spectrum, NIRS could also be used for the assessment of CytOx oxygenation, but the near-infrared absorption spectra of the two CytOx states overlap those of the hemoglobins, and the concentration of the latter is significantly greater [93,98,99,100,101]. Myoglobin is also involved in infrared light absorption in muscle: its oxygenated and deoxygenated spectra are identical to those of hemoglobin [14,96,102], so that muscle oximetry actually measures the oxygenation state of both hemoglobin and myoglobin molecules [14,103]. The evaluation of CytOx concentration and muscle oxygenation are not discussed in the current review.

As claimed in the current review, the primary problem of oximetry is the discrimination between the absorption and scattering effects in the attenuation measurement. The different light *absorption* for two wavelengths is the *signal*, while attenuation due to light-loss G and elongation of the path-length *l* (Equation (3)) is the primary *noise*. In arterial pulse oximetry the discrimination between the absorption and scattering effects is achieved by measuring the light attenuation difference between the end-diastole and the end-systole, and assuming that the systolic light attenuation change is mainly due to absorption, neglecting attenuation changes due to the scattering (Equations (7) and (8)). In NIRS, reflected/transmitted light intensity is measured as a function of time or distance from the light-source, and the diffusion equation, presented below, is utilized for deriving two equations that enable the determination of the two unknowns, the absorption constant µ_a_ and the reduced scattering constant µ_s_’. The diffusion equation enables the derivation of the photon fluence rate φ(r,t) that is reflected/transmitted from the medium surface [96,97], provided that the detailed structure and composition of the tissue are given. Since the real tissue structure is complex and unknown, solution of the diffusion equation for a specific tissue is impractical, and a simple model, simulating the real situation, must be used for NIRS measurements.

In general, the model includes light with a narrow cross-section emitted into a semi-infinite homogenous medium that simulates the biological tissue, and a detector that measures the intensity I_R_(ρ,t) of the reflected light, which is a function of its distance ρ from the light-source and of the time t (if the emitted light is time-dependent). I_R_(ρ,t) can be obtained by means of the diffusion equation [96,97,104,105] that relates the rate of change of φ(r,t) to the absorption and scattering coefficients of the medium and the photon emission source intensity.

Solutions satisfying the diffusion equation for the semi-infinite medium model with uniform absorption and scattering coefficients have been determined for three categories of light intensity dependence on time: short impulse, sinusoidally modulated intensity and constant intensity. For each sort of time-dependence, the corresponding solution of the reflected light intensity R(ρ,t) is related to the absorption constant, µ_a_, and the reduced scattering coefficient, µ_s_’. In accordance with the different time-dependences of the illumination, three modalities of NIRS have been developed, sorted as constant intensity (CW) spatially-resolved, time-resolved or frequency-resolved spectroscopy, where each modality makes use of the dependence of R(ρ,t) on the light source-detector distance, temporal dispersion of short light pulse or phase change of high-frequency intensity-modulated light, respectively [96,97,106].

Time-resolved spectroscopy is based on a short impulse emitted into a tissue volume and scattered from the tissue as a broadened pulse, due to different pathlengths between light entrance and exit for different photons. Since the speed of light in tissue is about 2 × 10^10^ cm/s, the time-of-flight of a photon for typical pathlengths of 2 to 10 cm is 100 to 500 picoseconds. In practice, the short impulse used in time-resolved spectroscopy has a width of a few picoseconds and the broadened reflected pulse demonstrates a time-width of several hundred picoseconds. For the semi-infinite medium model, a solution of the diffusion equation R(ρ,t) can be obtained [96,104,106,107], providing relationship between R(ρ,t) and µ’_s_ and µ_a_. By fitting the theoretical solution to the dependence-on-time of the measured reflected light, µ_s_’ and µ_a_ can be determined.

In the frequency-resolved spectroscopy µ_s_’ and µ_a_ can also be determined by solving the diffusion equation for the sinusoidal illumination function. The two unknowns, µ_s_’ and µ_a_, can be derived from the amplitude modulation and the phase shift of the reflected light, obtained from the diffusion equation solution [104,106]. Typical values of the modulated light frequency in frequency-resolved spectroscopy are 300–400 MHz.

NIRS measurement of blood oxygenation in the brain is based on light scattering from relatively deep tissue, 1–2 cm below the surface, which necessitates a source-detector distance of 2–4 cm, corresponding to a pathlength of 10–20 cm. Because the light irradiation level into the brain is limited, the transmitted/reflected light has a low intensity and very sensitive detectors must be used. Considering the high time-resolution that is required in the time- and frequency-resolved NIRS for measuring the broadening of the ultrashort pulses or the phase-shift of the very high frequency irradiation, sophisticated techniques of elevated cost must be used. However, sustained technological progress is expected to reduce the costs of the sophisticated time- and frequency-resolved NIRS devices.

In the CW spatially-resolved spectroscopy, reflected light in several wavelengths is measured in several light-sources/detector distances. The solution of the diffusion equation for the semi-infinite medium model relates R(ρ) to some function of the two unknowns, µ_a_ and µ’_s_, and in order to obtain µ_a_ separately, several equations have been proposed [96,97,107,108]. By applying the diffusion equation in two wavelengths, two equations for µ_a_(λ) at two wavelengths can be determined and [HbO_2_] and [dHb] can be derived from the two equations, based on Equation (4):

εC = µ_a_(λ) = ε_O_(λ)[HbO_2_] + ε_D_(λ)[dHb] (19)

The tissue oxygen saturation, StO_2_, can be determined from the [HbO_2_] and [dHb] values, as StO_2_ = [HbO_2_]/([HbO_2_] + [dHb]).

StO_2_ can also be obtained by the spatially-resolved NIRS by differentiating the diffusion equation with respect to ρ, which provides a relationship between the gradient of R(ρ) and µ_a_µ_s_’ [96,107,108,109,110]. Since µ_a_ = εC

µ_a_µ_s_’ = µ_s_’εC = ε_O_StO_2_µ_s_’C + ε_D_(1-StO_2_)µ_s_’C, (20)

See Equation (19). Measuring µ_a_µ_s_’ with two wavelengths and assuming that the difference in C between the two wavelengths is negligible and that µ_s_’ decreases slightly and linearly with the wavelength [107,110], StO_2_ can be derived from Equation (20) in two wavelengths, using an algorithm that is similar to that for SaO_2_ measurement by pulse oximetry, see Equations (7) and (8).

The accuracy of the NIRS techniques has been challenged on theoretical grounds, as the solution of the diffusion equation is based on a simplistic homogeneous semi-infinite medium model. The model does not reflect the real complex cerebral tissue structure, covered by cerebral-spinal fluid and bone, which also varies between subjects [96,97,111,112]. Fantini et al. [105] also raised an argument against the applicability of the diffusion equation to the *semi-infinite* model that simulates the tissue in noninvasive NIRS measurements, where the light-source and the detector are placed on the skin surface. The diffusion equation is an approximation that is only appropriate for measurements performed deep inside the bulk medium [105,113].

StO_2_ was also obtained by means of a *single* light-source/detector distance, making use of the modified Beer–Lambert equation (Equation (3)) in two or more wavelengths, *without utilizing the diffusion equation and the semi-infinite medium model*. In that technique, the scattering light-loss G and the absorption by skin blood and pigmentation are not canceled out and the cerebral StO_2_ is obtained by calibration with saturation measurements in the jugular vein and arterial blood [114,115,116]. The calibration of the cerebral StO_2_, which is a composite of oxygen saturation in arterial and venous blood, is based on the following assumptions: the *venous* blood oxygen saturation in a cerebral site is equal to that of the jugular vein, which drains blood from that cerebral site; the saturation in cerebral arterial blood is equal to that of the systemic arterial blood; the arterial blood to venous blood proportion in the cerebral tissue is a known ratio (30:70 or 25:75).

NIRS devices with a single light-source/detector distance are sensitive to the interference of extracerebral blood, while multi-distance NIRS devices can remove contamination from the extra-cranial circulation [117,118]. Some commercial devices only use a single pair of optodes separated by a distance of about 40 mm, which measures absorption in both cerebral and extracranial tissue, but adds measurement at a distance of about 20 mm, which aims to subtract the absorption signal of the extracranial tissue from the former absorption measurement. Measurements of cerebral StO_2_ by three commercial NIRS devices, with different values of extracerebral tissue perfusion (exploiting scalp ischemia), showed that the spatial resolved technique was not effective in isolating the absorption in the cerebral tissue [119].

Constant intensity illumination with a single light-source/detector distance was also used for measuring c*hanges* in tissue oxygenation by making use of the modified Beer–Lambert law (Equation (3)) and avoiding the use of the diffusion equation and the semi-infinite medium model. Temporal changes in [HbO_2_] and [dHb], induced by functional task, such as respiration or venous or arterial occlusion, can be derived from the corresponding temporal changes in light attenuation [96,97,101,107,120,121], assuming that the effect of light-loss G by scattering is the same for the two states and the attenuation difference between the two states substantially originated from absorption. The process of quantitative assessment of Δ[HbO_2_] and Δ[dHb] from the difference between the transmitted/reflected light intensity in the two states, I_1_ and I_2_, is similar to that of SaO_2_ measurement by pulse oximetry (See Equations (7) and (8)):

ln(I_1_/I_2_) = εΔC*l* = (ε_O_Δ[HbO_2_] + ε_D_Δ[dHb])*l*. (21)

Measurement of ln(I_1_/I_2_) in two wavelengths provides two equations with two unknowns, Δ[HbO_2_] and Δ[dHb], that can be solved for the two unknowns. The pathlength *l* for the two wavelengths can be obtained from the distance between the two optodes and the differential pathlength factor DPF. The DPF has been determined from time-resolved NIRS or by measuring the mean time-of-flight of ultrashort pulses of light propagating through the tissue [96,97,107,112,122], but since the variability among patients is large [96,120], the measurement accuracy is low. Nevertheless, *qualitative* information on temporal changes of [HbO_2_] and [dHb] due to functional tasks is commonly used in functional NIRS (fNIRS).

Cerebral fNIRS measures temporal changes of infrared light intensity transmitted through the brain, yielding temporal changes of [HbO_2_] and [dHb] in the cerebral tissue that follow functional tasks. The latter is originated from cerebral neural activity that results in a local increase in tissue metabolism and oxygen consumption, followed by vasodilatation and increased blood flow (neurovascular coupling) [123,124,125,126,127].

In cerebral fNIRS, one or a number of non-invasive skull probes are used to evaluate the increase in brain activity at specific locations in the cerebral cortex. Functional near infrared imaging (fNIRI) refers to the simultaneous measurement of local temporal oxygenation changes in multiple sites in a large area. In fNIRS and fNIRI, CW NIRS devices are commonly used, due to their small size, suitable time resolution and low-cost, despite the inability of the technique to quantify changes in [HbO_2_] and [dHb] in absolute terms. The determination of the brain activity location by fNIRS and fNIRI is of low spatial resolution (as compared to other brain imaging modalities, such as fMRI), because of the strong scattering of light in tissue and the long pathlength in NIRS [125,126].

Multi-channel NIRS, followed by image reconstruction, provides a two-dimensional image of the cerebral oxygenation level. A further advancement of fNIRI is the three-dimensional presentation of the cerebral oxygenation—diffuse optical tomography (DOT), which includes the ability to measure light after transmission through several overlapping light-source/detector distances, in each of the multiple sensors. The assessment of the oxygenation dependence on tissue depth is based on the fact that greater distance between the optodes is associated with deeper tissue pathlengths [125,126,127]. The limited number of light-sources and detectors sets a trade-off between resolution and measurement region area. DOT measurements on the whole cerebral cortex, with relatively high resolution, have been performed on newborns and infants, primarily for neurodevelopmental assessment and detection of neurodevelopmental disorders [126,128,129,130].

### 5.2. Validation and Accuracy

At present, the primary implementation of NIRS, in both clinical diagnosis and research, is in the assessment of tissue oxygenation in cerebral tissue, which is directly related to cerebral blood perfusion [131,132,133,134]. Timely assessment of the latter is important in critically ill patients and patients undergoing major surgical operations, because of their increased incidence of adverse outcomes, particularly neurological complications that may occur due to cerebral hypo-perfusion. Hence, a reliable noninvasive technique for StO_2_ assessment is expected to be beneficial for the improvement of patient treatment and outcomes in perioperative and intensive care medicine.

A major difficulty of validating the cerebral oxygenation NIRS technology is the absence of acceptable and reliable methods for the measurement of cerebral tissue oxygenation to compare NIRS with [135]. In several studies, NIRS-derived cerebral StO_2_ readings were compared to oxygen saturation in the jugular vein (SjO_2_), which drains venous blood from one of the brain hemispheres. Both NIRS StO_2_ and invasive SjO_2_ decreased during induced hypoxia, though the deviations between the values of the two parameters were significant [100,135]. In the study of Rosenthal et al. [136], NIRS StO_2_ correlated with SjO_2_ when the latter was measured in blood drained from the hemisphere with the NIRS probe site (r = 0.60, *p* < 0.001), but not with SjO_2_ measured in the jugular vein that drains blood from the contralateral hemisphere.

As StO_2_ is the mean oxygen saturation in the entire blood in tissue, arterial and venous, cerebral StO_2_ readings were compared to a weighted mean of oxygen saturation in blood extracted from the jugular vein and a systemic artery (SavO_2_) [114,115,135], and the comparison rendered smaller deviations than those found for SjO_2_. The weight of the cerebral arterial and venous blood is generally based on the assumption that the venous-to-arterial blood volume ratio of the cerebral tissue is 70:30, as was found by PET measurements [137]:

SavO_2_ = 0.3SaO_2_ + 0.7SjO_2_. (22)

SaO_2_ is generally measured by pulse oximetry or by extracting blood from an artery utilizing a co-oximeter; SjO_2_ is measured invasively by means of a co-oximeter in extracted blood from a jugular vein.

Validation studies of commercial cerebral oximeters were performed on healthy volunteers or patients with jugular catheterization, after calibrating NIRS StO_2_, by invasive measurements of SavO_2_ on a different group of healthy volunteers. In these studies [114,115,116,138,139,140], the StO_2_/SavO_2_ correlation coefficient values were 0.7–0.9, the bias values (mean difference between their individual values) were 0.5–1.2%, and the standard deviation values were 3–5.4%.

Bickler et al. [100] compared cerebral StO_2_, measured in healthy volunteers by five commercial cerebral oximeters, with invasive SavO_2_ during induced hypoxemia. The mean bias values for the different devices were −1.13–2.84 and the standard deviation was 3.92 for one of the devices and 6.27–9.72 for the others. While the cerebral oximeters were found to be effective in detecting the induced desaturations, the significant variation in baseline readings among the five devices demonstrated inaccuracy in *absolute* cerebral StO_2_ measurement by the current NIRS devices. Furthermore, the great variation in cerebral StO_2_ values among the healthy subjects limits the ability of the current non-invasive cerebral oximeters to yield a definite threshold value of the cerebral StO_2_ that might lead to tissue damage [100].

La Cour et al. [135] reviewed available in vivo validation studies, which compared noninvasive cerebral NIRS-based StO_2_ to SavO_2_ in pediatric and adult populations, and found poor agreement in both adults and children: the difference between the limits-of-agreement values was of about 13%. (The limits-of-agreement are the mean of the differences between the two parameters ±1.96 standard-deviations). A comparison of StO_2_ to SjO_2_ rendered lower agreement: the difference between the limits-of-agreement was about 18%. The poor agreement between cerebral StO_2_ and SavO_2_ can be attributed to differences of the arterial-to-venous blood-volume ratio between subjects and differences within subjects, due to changes in hypoxia level. In addition, the jugular vein drains blood from the whole brain hemisphere, while the non-invasive NIRS only measures saturation in the cerebral tissue, and StO_2_ in the cerebral and non-cerebral tissues are not necessarily equal [100,135].

The partial pressure of oxygen in a tissue (PtO_2_) is a physiological parameter that is related to StO_2_ through the oxygen–hemoglobin dissociation curve. Cerebral PtO_2_ is measured invasively in the brain tissue of patients with traumatic brain injury for clinical purposes, and in a number of studies, its association with cerebral StO_2_ was examined. Despite the relationship of both parameters to the balance between tissue oxygen delivery and consumption, only poor or no correlation between cerebral StO_2_ and PtO_2_ was found [117,118,141,142], probably because of the nonlinear relationship between StO_2_ and PtO_2_ in the blood (the oxygen–hemoglobin dissociation curve).

The precision of NIRS devices, i.e., the variation of repeated measurements at the same site, was also evaluated in a number of studies on neonates, rendering values of 5.2% [111] and 2.0% on term and 4.2% on preterm neonates [143]. In a recent study [144] on preterm neonates, a precision value of 2.64% was found, but some part of the StO_2_ variability was attributed to spontaneous hemodynamic fluctuations. Using methods for reducing the fluctuations’ effect, an improved precision value of 1.85% was achieved.

The validation studies dealt with in the current subsection, and evaluated the accuracy of cerebral StO_2_ readings measured in *absolute terms* by NIRS cerebral oximeters, based on CW spatially-resolved spectroscopy and calibration by invasive SjO_2_ measurements. In those studies, the accuracy of the commercial devices based on the NIRS technique was found to be low, and the inter-subject and inter-device deviations were substantial. Nevertheless, qualitative information on cerebral StO_2_ can be obtained by NIRS, and changes in cerebral StO_2_ as a function of time can be detected reliably, as will be described in the following subsection.

### 5.3. Clinical Applications

The potential benefit of NIRS cerebral StO_2_ readings for clinical treatment has been evaluated in a number of studies, in particular in randomized intervention studies on patients undergoing major surgical operations. In the latter studies, in the intervention group, cerebral StO_2_ was monitored during the surgical operation by NIRS, and supplemental oxygen dosage was adjusted according to StO_2_ level, while in the control group, the treatment was not directed by NIRS readings. In several studies, the intervention group demonstrated a decreased incidence of adverse events compared with the control group [145,146,147], while in similar studies [148,149,150], the NIRS-guided intervention was not associated with improved patient outcomes. Yu et al. [151], in a systematic review, inferred low quality of evidence for the beneficial effect of cerebral NIRS monitoring on postoperative adverse events, such as stroke, delirium or death, due to a low number of events and wide confidence intervals.

The benefit of NIRS cerebral StO_2_ measurements for clinical treatment in preterm neonates has also been evaluated in a randomized intervention clinical trial, performed in eight neonatal intensive care units [152,153,154]. In the intervention group, the burden of hypoxia was significantly lower than that in the control group, but no decrease was found in all-cause mortality, brain injury score and other clinical outcomes [152,153]. No difference in neurodevelopment outcomes at two years of corrected age was found between the two groups [154].

In observational studies, decreased perioperative cerebral StO_2_ was associated with poor patient outcomes after a major surgery [155,156]. In a recent systematic review [133], an association between low cerebral StO_2_ and higher incidence of delirium was found in four studies in critically ill patients, compared to controls [157,158,159,160], but only in two of them [159,160] was the relationship statistically significant.

Continuous assessment of brain tissue blood flow and oxygenation of brain tissue is clinically important for traumatic brain injury (TBI) patients, for whom rapid diagnosis is essential for the timely restoration of brain metabolic function and the avoidance of secondary brain injury. In order to identify episodes of cerebral ischemia and hypoxia that can deteriorate patients’ clinical state, several *invasive* techniques are being used to measure physiological markers, such as intracranial pressure and PtO_2_. The opportunity to replace those invasive modalities with noninvasive ones has motivated observational studies that examined the association of CW NIRS StO_2_ monitoring in adult TBI patients with their functional outcome and with physiological-neurological parameters [117,118,161]. Several studies showed that the detection and evaluation of cerebral hypoxia by NIRS were related to increased mortality or poor functional outcome, but the evidence was relatively weak, and additional research is needed before NIRS-based techniques are routinely utilized as a reliable clinical tool.

An interesting application of NIRS StO_2_ monitoring is the assessment of cerebral autoregulation (CAR)—the ability of the brain to keep cerebral blood flow (CBF) relatively constant during changes of mean arterial blood pressure, as long as the latter is maintained within defined limits (Figure 5) [162,163,164]. CAR protects the brain from hypo-perfusion caused by low perfusion-pressure and from hypertension episodes that can lead to elevated intracranial pressure and to damage to the micro-vessels. In adverse events such as traumatic brain injury, the CAR mechanism might be compromised, potentially leading to an exacerbation of the patient’s condition. Evaluation of CAR can be done from the relationship between the temporal changes in CBF and those in mean arterial blood pressure (MAP)—a positive correlation between them indicates impaired CAR, while intact autoregulation leads to a low correlation [118]. However, since CBF cannot be measured directly, surrogate parameters are being used, such as intra-cranial pressure, measured invasively, or trans-cranial Doppler velocity, measured in the middle cerebral artery. A number of quantitative CAR indices, based on those surrogate measurements of CBF, have been developed for CAR assessment [165]. Cerebral NIRS-derived StO_2_ has also been used as a surrogate for spontaneous CBF changes, as tissue oxygenation is directly related to tissue perfusion, and continuous NIRS StO_2_ measurement is simpler to use than continuous trans-cranial Doppler measurement.

NIRS-derived CAR indices have been compared to those based on trans-cranial Doppler monitoring, and correlation values of 0.40–0.81 were found between indices obtained by the two methods [117,118,166,167,168,169,170]. In a study performed on children during cardiopulmonary bypass [171], high values of the NIRS-derived CAR index were associated with hypotension. The latter study also found that the lower MAP limit of the CAR curve (Figure 5) can be determined from the NIRS CAR index.

The correlation between the two sets of CAR indices, obtained by trans-cranial Doppler velocimetry and by NIRS, is significant but low, and only allows validation in qualitative terms of the NIRS-based CAR assessment. As was described above, validation studies of the cerebral oximeters reported low accuracy of the technique and substantial inter-subject and inter-device deviations when StO_2_ readings were measured in *absolute terms*, while *changes* of cerebral StO_2_ can be obtained qualitatively.

The review was focused on oximetric measurements on humans, but cerebral oxygenation has also been measured by NIRS and fNIRS on animals, both for physiological and pathological animal research and for the assessment of the techniques. Some references [172,173,174,175,176], not representative of studies describing cerebral NIRS and fNIRS measurements on animals, were presented.

## 6. Conclusions

Due to the paramount physiological importance of adequate oxygen supply to the tissue, clinical evaluation of blood oxygenation in arteries and veins is essential, motivating the development of clinical techniques for the measurement and monitoring of blood oxygenation in arteries, in veins or in both arteries and veins in the tissue. The non-invasive nature of light propagation through tissue and the different spectra of oxygenated and deoxygenated hemoglobin stimulated the development of several oximetric techniques for the assessment of oxygen saturation and concentrations of oxygenated and deoxygenated hemoglobin, parameters that provide information on blood oxygenation. The current review presents the main oximetric techniques for the assessment of blood oxygenation by measurements of attenuation in tissue of light comprising two wavelengths, utilizing the different absorption spectra of oxygenated and deoxygenated hemoglobin. The various oximetric techniques enable the evaluation of oxygenation in arterial or venous blood or in the entire blood in tissue, in finger, peripheral muscle, brain or retina—tissues that are accessible to light in the visible or infrared regions.

The primary challenge of oximetry is the discrimination between the effects of absorption by hemoglobin (signal) and scattering by tissue elements (noise) in the attenuation process, and several mechanisms have been created to isolate the absorption effect. Currently, only two techniques, pulse oximetry for the measurement of oxygen saturation in arterial blood, and NIRS, for the assessment of tissue oxygenation in cerebral and muscle tissue, are being used in clinical practice, and only pulse oximetry has been widely accepted as a valid diagnostic tool. The use of photoplethysmography, for the isolation of absorption in arterial blood, enabled pulse oximetry to provide arterial blood oxygen saturation measurements with reasonable accuracy. The accuracy of pulse oximetry seems to be sufficient for most clinical requirements, though in patients under oxygen supplementation, preterm neonates in particular, greater accuracy is required to avoid excessive oxygenation. In regard to NIRS, it seems that significant improvement in its technical aspects is required in order to obtain *reliable absolute* measurement of cerebral oxygen saturation, a prerequisite for a medical device to be widely accepted as a clinical tool.

## Abbreviations

CaO_2_	oxygen content in arterial blood
[Hb]	hemoglobin concentration in blood
HbO_2_	oxygenated hemoglobin
dHb	deoxygenated hemoglobin
SO_2_	oxygen saturation in blood vessels, arteries or veins
SaO_2_	arterial oxygen saturation
SpO_2_	arterial oxygen saturation measured by pulse oximetry
PaO_2_	arterial oxygen tension
SvO_2_	oxygen saturation in venous blood
StO_2_	oxygen saturation in tissue blood
SjO_2_	oxygen saturation in the jugular vein
SavO_2_	weighted mean of oxygen saturation in blood in the jugular vein and in a systemic artery
µ_a_	absorption constant
µ_s_	scattering coefficient
µ_s_’	reduced scattering coefficient
g	anisotropy
ε_O_	extinction coefficients of HbO_2_
ε_D_	extinction coefficients of dHb
ε	the mean extinction coefficient of blood in the tissue vessels, arteries and veins
C	hemoglobin concentration
I_0_	incident light intensity
I_t_	transmitted light intensity
d	emitter-detector distance
*l*	mean optical path-length
DPF	differential pathlength factor
G	light-loss due to tissue scattering
OD	optical density
PPG	photoplethysmography
I_D_	The maximal value of the PPG pulse
I_S_	The minimal value of the PPG pulse
AC	the difference between the maximal and minimal values of the PPG pulse
DC	the mean light transmission during the pulse
λ	wavelengths
ΔC	the maximal hemoglobin concentration increase in the tissue during systole
R	ratio of ratios
NIRS	Near infrared spectroscopy
TOI	tissue oxygenation index
rSO_2_	regional oxygen saturation
CytOx	cytochrome oxidase
I_R_(ρ,t)	the intensity of the transmitted/reflected light in NIRS
ρ	the distance from the light-source
t	time
CW	constant wave/intensity
fNIRS	functional NIRS
fNIRI	functional near infrared imaging
DOT	diffuse optical tomography
PtO_2_	oxygen partial pressure in a tissue
TBI	traumatic brain injury
CAR	cerebral autoregulation
CBF	cerebral blood flow
MAP	mean arterial blood pressure
